# How autism shows that symptoms, like psychiatric diagnoses, are 'constructed': methodological and epistemic consequences

**DOI:** 10.1007/s11229-020-02988-3

**Published:** 2021-01-23

**Authors:** Sam Fellowes

**Affiliations:** Department of Politics, Philosophy and Religion, County South Lancaster University, Lancaster, LA1 4YL UK

**Keywords:** Psychiatric symptoms, Psychiatric diagnoses, Realism, Constructivism

## Abstract

Critics who are concerned over the epistemological status of psychiatric diagnoses often describe them as being constructed. In contrast, those critics usually see symptoms as relatively epistemologically unproblematic. In this paper I show that symptoms are also constructed. To do this I draw upon the demarcation between data and phenomena. I relate this distinction to psychiatry by portraying behaviour of individuals as data and symptoms as phenomena. I then draw upon philosophers who consider phenomena to be constructed to argue that symptoms are also constructed. Rather than being ready made in the world I show how symptoms are constructs we apply to the world. I highlight this with a historical example and describe methodological constraints on symptom construction. I show the epistemic problems with psychiatric diagnoses are also applicable to symptoms. Following this, I suggest that critics of psychiatric diagnoses should extend their criticism to symptoms or, if they still believe symptoms are relatively epistemologically unproblematic, should rethink their concerns over psychiatric diagnoses.

## Introduction

Critics often claim that most or all currently employed psychiatric diagnoses are constructed, arbitrary, an invention or made-up. They argue that those psychiatric diagnoses should be abandoned. They vary over whether psychiatric diagnoses should be fully abandoned, being replaced with some alternative which are not psychiatric diagnoses, or if they should be replaced with superior psychiatric diagnoses. Many, though not all, such critics have much less concern about psychiatric symptoms. These critics do not argue that most or all symptoms should be abandoned, to be either replaced with some alternative which are not symptoms or replaced with alternative, superior symptoms. Such critics of psychiatric diagnoses typically believe that people do actually exhibit symptoms (for example Boyle 1990, p. 166; Cushing 2013, p. 39; Cuthbert 2014, p. 32; Johnstone 2018, p. 39; Kinderman et al. 2013, p. 3; Kirk et al. 2015, p. 67; Read 2004a, p. 48; Vanheule 2017, p.162). For example, it is generally accepted that some people do actually exhibit low social skills and low eye contact. However, in this article I will argue that psychiatric symptoms face many of the same criticisms that are levelled at psychiatric diagnoses. In line with common criticisms of psychiatric diagnoses, symptoms should not be understood as ready-made, out there in nature ‘things’ which people exhibit. Rather, symptoms have to be assigned to people. Psychiatric symptoms, like psychiatric diagnoses, could also be considered as ‘constructed’ or ‘invented’. This means that psychiatric diagnoses and symptoms have a more similar epistemic status than is typically presumed. I will show how this has consequences for the epistemic status of both symptoms and diagnoses.

I will develop a new conceptual understanding of symptoms. I demarcate between, firstly, individual instances of behaviour which vary from one another in how they specifically manifest and, secondly, symptoms which abstract away those specific details. The behaviour which an individual actually exhibits is not the same as the symptoms that they are assigned because the behaviour has specific details which are missing from the abstract symptom. To make this argument, I draw upon a distinction employed by some philosophers of science, that of *data* and *phenomena*. *Data* is the product of specific causal factors which are present in specific experimental set-ups, such as the ambient air temperature on a particular day. These can vary from one experimental setup to another. In contrast, *phenomena* abstracts away those specific details to create a more generalised scientific concept which is applicable to multiple experimental setups or real world settings rather than being tied to specific factors present in specific situations. For example, the melting point of lead is considered to be 235 °C even though the exact figure measured can vary due to the environmental conditions present during specific measurements. Following this distinction I portray behaviour as data and symptoms as phenomena. Behaviour, like data, are tied to specific instances whereas symptoms, like phenomena, are models which abstract away those specific details. I highlight this distinction with an historical example and then, outline constraints on formulating symptoms from behaviour.

Finally, I argue that conceptualising symptoms as phenomena has an important consequence for the status of symptoms. Phenomena should be conceptualised as constructed by scientists rather than as coming ready made by the world. Following this, symptoms should also be seen as constructed by psychiatrists rather than being ready made. This has epistemic consequences for both symptoms and diagnoses. Critics often associate construction with arbitrariness and bad science (Bentall 1992, p. 24; Burrows 2010, p. 252; Tucker 1998, p. 159, for further discussion from non-critics see Beebee and |Sabbarton-Leavy 2010, p. 16; Meehl 1995, p. 268). However, I show that symptoms, like diagnoses, are constructed. Critics can hold different reasons for taking construction to entail that psychiatric diagnoses are flawed so I will be more specific in my discussion. I show that many arguments which critics use against psychiatric diagnoses are also applicable to symptoms. I then outline three options for critics of psychiatric diagnoses. Firstly, critics who are concerned over psychiatric diagnoses because they are constructed might have similar concerns about symptoms. Secondly, a critic of psychiatric diagnoses who thought symptoms, despite being constructed, are relatively unproblematic might reconsider their concern over psychiatric diagnoses. Thirdly, a critic could argue that symptoms are constructed in an unproblematic manner whereas psychiatric diagnoses are constructed in a problematic manner. My primary aim in this paper is to present a challenge to the critics of psychiatric diagnoses. Critics need to show why psychiatric diagnoses (or particular psychiatric diagnosis) are constructed in a problematic manner or either also reject symptoms (since they are constructed) or accept psychiatric diagnoses (even though they are constructed). More tentatively, I finish by sketching my preferred response to the dilemma I have posed. I suggest the best way to respond to the claim that symptoms and diagnoses are both constructed is to argue that construction need not lead to scepticism. I will argue that we should accept both psychiatric diagnoses and symptoms in principle, and in actuality many currently employed psychiatric diagnoses and symptoms should be accepted. Both psychiatric diagnoses and symptoms are constructed, but construction need not be a barrier to scientific worth in psychiatry.

## Construction in psychiatry

Critics often claim that (some or all) psychiatric diagnoses are “arbitrarily constructed” (Burrows 2010, p. 252), “constructed” (Gains, 1992 p. 4; Summerfield 2001, p.95), “arbitrary” (Cushing 2013, p. 38; Horwitz 2002, p. 5) an “invention” (Read 2004b, p. 21; Summerfield 2001, p. 95), “made-up” (Watson 2019, p. 2). They are also considered to be often formulated on harmful extra-scientific factors (Cooper 2005, p. 150; Jablensky 1999, p. 138; Poland 2014, p. 35) or to be epistemologically weak (Cooper 2005, p. 150; Murphy 2006, p. 10; Poland 2015, p. 37). These critics come from a variety of fields, such as psychiatry, philosophy of psychiatry, psychotherapy, sociology, history and disability studies. They hold a significant diversity of implicit and explicit standpoints. For convenience, I shall use the general term ‘constructed’ in what follows to cover these concerns. There are various ways in which a psychiatric diagnosis can be considered ‘constructed’ which I shall outline below.

Firstly, psychiatric diagnoses can be seen as always taking a constructed form or can be seen as potentially non-constructed depending upon if there is a target for them to describe. Psychiatric diagnoses can be seen as necessarily constructed because they lack a target in the world which they could reflect or resemble. Reality is seen as not taking a form whereby people fall into groupings which psychiatric diagnoses could describe. Alternatively, psychiatric diagnoses can be seen as not necessarily constructed because there is a target which they could describe. People, or particular aspects of people, sometimes take a form which is amenable to description by psychiatric diagnoses. In this case then when a critic claims psychiatric diagnoses are constructed they mean that most or all currently employed psychiatric diagnoses fail to describe any suitable target. However, it is believed to be possible to produce alternative psychiatric diagnoses which are not constructed.

Secondly, constructs can be seen as scientifically problematic or as scientifically non-problematic. By scientifically problematic I mean non-scientific, weak science or bad science. Construction can be considered scientifically problematic if the construct is seen as too greatly the product of a decision making process rather than scientific evidence. Various views can be held over what constitutes scientific evidence and over what degree of decision making is acceptable given any particular level of scientific evidence available (decision making might be seen as more acceptable, or as less acceptable, when higher levels of scientific evidence are present). A critic would claim psychiatric diagnoses are constructed in a problematic manner if they think they exceed an unacceptable level of decision making. In contrast to this approach, it is possible to consider construction as scientifically acceptable whereby construction is seen as fully compatible with good science.

Following these demarcations, there are critics who think that psychiatric diagnoses are necessarily constructed and that construction is scientifically problematic. These critics think that we should not only stop using currently employed psychiatric diagnoses but also not attempt to develop alternative, superior, non-constructed psychiatric diagnoses (since a non-constructed psychiatric diagnosis is impossible). We should simply stop using psychiatric diagnoses altogether (Boyle 1990, p. 166; Bentall 1992, p. 34; Cromby et al. 2015, p. 116; Johnstone 2018, p. 39; Kinderman et al. 2013, p. 3; Kirk et al. 2015, p. 67; Read 2004a, p. 48; Runswick-Cole 2016, p. 27). Then there are critics who think that currently employed psychiatric diagnoses are constructed, but also that alternative psychiatric diagnoses could be non-constructed, and also that construction is scientifically problematic. These critics typically call for abandoning currently employed psychiatric diagnoses and replacing them with new, superior ones. Some such critics think superior psychiatric diagnoses should still be DSM-style categorical, polythetic psychiatric diagnoses (Cooper 2005, p. 53; Murphy 2006, p. 224) and others think that we should move to dimensional psychiatric diagnoses (Cuthbert and Insel 2013, p. 3; Lilienfeld 2014, p. 131).

Analogous concerns about construction are sometimes raised about symptoms. Symptoms can be seen as necessarily constructed or as potentially non-constructed. In the latter case, currently employed symptoms could be seen as never, rarely, often or always constructed. Additionally, constructed symptoms can be seen as problematic or non-problematic. Whether they are described as constructed or not it is certainly true that there is concern over the scientific adequacy of various symptoms. For example, there are concerns over the manner in which hallucinations are defined (see Parnas and Sass 2008, p. 249; Campbell 2002, p. 96). As a second example, there are concerns over how notions of empathy are understood (see Ratcliffe 2014, p. 275; Slaby 2014, p. 255).

Having demarcated various views on construction I now outline the target of my article. I focus upon critics who are concerned about psychiatric diagnoses but who have much less concern about symptoms. There are many critics who are concerned over psychiatric diagnoses being constructed but are not concerned, or have much less concern, over symptoms being constructed. It is widely accepted, even by critics of psychiatric diagnoses, that many diagnosed people exhibit symptoms (for example, Boyle 1990, p. 166; Cushing 2013, p. 39; Cuthbert 2014, p. 32; Kirk et al. 2015, p. 67, Vanheule 2017, p. 206). Few people doubt, for example, that most people who meet the diagnostic criteria of autism do actually have low social skills and exhibit low eye contact. Eyes are real parts of the world. Where eyes look and for how long are measurable facts. Similarly, the manner in which people socialise is in some sense real and is potentially measurable. That diagnoses are considered more problematic than symptoms can be highlighted through considering how some critics propose abandoning psychiatric diagnoses to instead focus on symptoms. For example, in an article calling for the abandonment of psychiatric diagnoses, Kinderman, Read, Moncrieff and Bentall still talk of “symptoms” and how a future psychiatry would focus upon things like “depressed mood, auditory hallucinations and intrusive thoughts” (2013, p. 2) rather than psychiatric diagnoses. Also, a critic of autism “propose[s] [to] focus instead on specific deficiencies, like sensory processing disorders, communication difficulties or food sensitivities and stop trying to cluster them together as something called ‘autism’” (Cushing 2013, p. 39). Also, a critic of schizophrenia writes that she does not deny that people exhibit many of the symptoms associated with schizophrenia, such as hearing voices and confused thinking. She believes psychiatrists “should devote enormous energy and resources to trying to understand why these phenomena occur and what variables influence them, but without inferring unsupported concepts like schizophrenia” (Boyle 1990, p. 166). Consider also how philosophers of psychiatry Cooper and Murphy argue that almost all psychiatric diagnoses are epistemologically problematic and in need of replacement (Cooper 2005, p. 150; Murphy 2006, p. 10) whereas they do not make similar claims about symptoms. Rather, they have concerns over a much smaller list of symptoms. Finally, projects like Research Domain Criteria (RDoC) hope that currently employed psychiatric diagnoses are reformulated into dimensional ones but notions that currently employed symptoms need replacing are near completely absent within the many publications which the RDoC project has produced. As the current director of RDoC writes, “[t]he concern about the current diagnostic environment has not been so much with the symptoms themselves” (Cuthbert 2014, p. 32). Explicit statements saying that symptoms are usually not constructed or are constructed in a scientifically unproblematic manner are absent from these sources but they seem to treat most symptoms as non-constructed and as scientifically unproblematic.

## The distinction between behaviour and symptoms

Psychiatrists typically see people as exhibiting symptoms. For example, autistic people are considered to typically exhibit symptoms like low social skills and repetitive behaviour whilst schizophrenic people are typically considered to exhibit symptoms like hallucinations and delusions. Symptoms are typically considered to be ways of behaving.^1^

In this paper, I will critique the notion that people straightforwardly *have* symptoms. Claiming that people have symptoms conceals some important distinctions which this paper brings out. It is more accurate to say that people behave in particular ways but are *assigned* symptoms.

To highlight the distinction between behaviour and symptoms I now show how two instances of behaviour can differ from one another yet still constitute an instance of the same symptom. Imagine two instances of the symptom low eye contact. Both instances will involve an abnormal lack of eye contact. However, the exact level of eye contact will likely differ in each case. So too will where each pair of eyes look and for how long. Specific details which are applicable to one instance of low eye contact are not applicable to the other instance. Additionally, various factors might influence the level of low eye contact given in each instance. For example, perhaps one person feels anxious and so exhibits even less eye contact than usual, whilst the second person is talking to a trusted family member so exhibits more eye contact than usual. Similarly, there are specific factors which vary between particular manifestations of low social skills. Instances of low social skills will also vary radically in the variety of possible words said, in the tone they are said and in the body language which they are accompanied by. There can also be many influences on when, how and to what degree social skills are exhibited, such as who is being spoken to, in what setting and for how long. Consider also the variety of possible hallucinations, the variety of causal factors on anxiety, and variety of possible content in repetitive thought.

In contrast, symptoms lack the detail which is specific to particular manifestations of behaviour. That detail has been abstracted away. Consequently, symptoms are much more generalisable. It is not the case that there is one symptom of low social skills for talking to your boss and a different symptom of low social skills for talking to your parents. Rather, such details are abstracted away. This means symptoms are more generalisable and applicable to multiple situations. They can be assigned to multiple people rather than just being tied to the specific details of a particular person. Two people can be considered to exhibit the same symptom despite exhibiting different behaviours.

Having outlined the distinction between behaviour and symptoms I now develop a conceptual understanding of symptoms. To do this I consider the distinction between data and phenomena.

## Are symptoms ready made or constructed?

To develop a conceptual understanding of symptoms, I draw upon a well-known distinction made by Bogen and Woodward in their seminal 1988 paper *Saving the Phenomena*. Traditionally, philosophy of science demarcates sharply between *data* and *theory*. Traditionally, *data* is seen as the product of experiments and theories explain that data. Bogen & Woodward argued this picture missed out an important intermediate step. They suggest the traditional picture of data and theories needs to be supplemented by *phenomena*, an additional step lying between them. The basis for this was the immense number of causal factors present when scientists perform experiments. They describe how

the outcome of any given application of a thermometer to a lead sample depends not only on the melting point of lead, but also on its purity, on the workings of the thermometer, on the way it was applied and read, on interactions between the initial temperature of the thermometer and that of the sample, and a variety of other background conditions (Bogen and Woodward 1988, p. 309).

The same experiment can result in the thermometer providing different figures. The exact temperature a thermometer registers depends on specific factors present in specific experimental conditions. Despite this, scientists still posit lead as having a melting point of 327.5 °C. Scientists thus believe that lead has the characteristic of having a specific melting point even though actual experiments can provide different figures. Consequently, Bogen and Woodward posited a notion of phenomena which is a more abstracted, more generalised notion than is given by individual experiments. Thus whilst different experiments provide results of 328.0 °C, 327.1 °C, 327.3 °C, etc., scientists might agree upon phenomena as 327.5 °C, a general figure abstracted away from specific experiments.

The motivation for formulating phenomena is that specific experimental setups are influenced by multiple causal factors but scientists may only be interested in certain causal factors. Bogen writes that “[d]ata typically result from complex, loosely connected, short-lived assemblies of causal factors… The causes that produce data sets are never exactly the same from one trial to another” (2010, p. 789). Many causes are not relevant to the phenomena, since, as Woodward comments, “in typical cases data are the result of many causal factors and at most [only] some of these will have to do with the phenomena of interest” (Woodward 2010, p. 167). Scientists typically want a general figure which is not tied to specific causal factors present in specific instances of the experiment. Depending on the topic of study, a scientist may not be interested in insignificant factors like variations in air temperature which have a minimal influence on results. Such causal factors are not considered the cause which determined the melting point of lead, rather, they are incidental factors which interfere with finding the correct figure. The true cause is considered to be the molecular structure of lead.

I employ Bogen and Woodward’s framework of data and phenomena to conceptualise the distinction I made between behaviour and symptoms. Behaviour should be understood as data and symptoms should be understood as phenomena. Instances of behaviour have, like data, details which are specific to particular instances. These are due to a multiplicity of causes being present. Symptoms, like phenomena, abstract away those details and so are generalisable, not being tied to specific instances.

I now consider the ontological status of phenomena. In their 1988 paper Bogen and Woodward hold a realist understanding of phenomena (1988, p. 337). They provide few details but seem to suggest phenomena are things in the world which scientists can discover. This realist approach faces the challenge of accommodating the abstract nature of phenomena. Additionally, Woodward has later developed his account of phenomena in two ways which potentially further challenge a realist understanding. Firstly, Woodward argues that “[d]ecisions to ignore or discard data play a central role in virtually all data-to-phenomena reasoning” (Woodward 2000, p. 177). Consequently, realist understandings need to accommodate choices being made over which results and causal influences to abstract away. Secondly, Woodward argues the phenomena which are formulated are relative to purpose (Woodward 2011, p. 174). This suggests that phenomena are not simply ‘things’ which are out there in the world independent of our interests. To further explore the ontological status of phenomena I draw upon Psillos for a realist understanding of phenomena and Massimi for a neo-Kantian understanding of phenomena.

Psillos takes a realist understanding of phenomena yet simultaneously rejects notions that phenomena are things which are out there in the world. He describes how “phenomena (e.g., the melting point of a substance or the path of a planet) are abstracted from the data by means of a number of sophisticated techniques based on a rather substantive set of assumptions and theories” (2004, p. 397). He describes how those assumptions and theories involve significantly unrealistic elements, giving the example of “*imponderable* fluids, *frictionless* planes, *ideal* gases, *perfectly* spherical objects” (Psillos 2011, p. 17). These are false assumptions since, for example, planes are not frictionless. They are idealisations which resemble “nothing in the physical world” (Psillos 2011, p. 8). He sees them as “abstract entities… not concrete objects” (Psillos 2011, p. 8). Psillos sees realism as based around what he calls the explanatory criterion (2011, p. 15). If a scientific theory (or more specifically an aspect of a scientific theory) contributes to the best explanatory framework for understanding the world then it should be considered real. Whether that thing is a concrete physical thing or is abstract is not relevant to that explanatory criterion (2011, p. 15). Consequently, phenomena can be understood as real despite them not being physical things.

Massimi has developed a neo-Kantian account which sees phenomena as made by scientists rather than ready-made by the world. She argues which phenomena scientists postulate depends upon how data is abstracted away. She gives the example of Galileo’s experiments, describing how

the goal of the inclined plane experiment was to extract from the appearance (motion of a bronze ball along an inclined plane) the property of uniform acceleration…. we should not think that what we observe, say, a free-falling object, is the rough-and-ready observable phenomena… If we stick to the level of observable[s]… Galileo may seem no more right than Aristotle (Massimi 2008, p. 25).

Galileo made observations of the ball and recorded various results. All these results would have been influenced by many different factors like friction and the varying surface of the wood. Consequently, when Galileo formulated the phenomena of uniform acceleration he had to abstract away all the non-uniformity. This means that, as Massimi suggests, “phenomena are something that… we *make* rather than something that comes to us as ready-made in nature” (Massimi 2008, p. 8 emphasis original). The actual speed at which the ball travelled in any given experiment was in some sense real and non-constructed. This, however, does not constitute uniform acceleration. Acceleration is not naturally uniform, rather, it only becomes so once the non-uniformity is abstracted away through constructing uniform acceleration. As, Massimi argues, “phenomena we infer depends on the way we have carved and ‘massaged’ those data” (2011, p. 104). Choices need be made about what to abstract away when formulating phenomena. For example, Galileo could have posited acceleration as non-uniform whilst an alternative figure for the melting point of lead could be posited if different aspects of the data were abstracted away.

Following these approaches I suggest that symptoms are not ready-made parts of the world. Symptoms can be understood, following Psillos’s realist notion of phenomena, as real models which have no counterpart in the actual world. Alternatively, they can be understood, following Massimi’s Kantian notion of phenomena, as being made by us, rather than as being ready-made in the world. On both of these approaches, symptoms should not be seen as ‘things’ which are out there in the world. As such, in what follows, I shall describe symptoms as being ‘constructed’. The word constructed is sometimes intended, including by critics of psychiatric diagnoses, to have negative epistemic connotations. I am not endorsing such connotations, rather, in Sects. 7.0 and 8.0 I shall consider whether ‘constructing’ symptoms entails negative epistemic connotations.

## Alternative ways to formulate symptoms

I will now show how symptoms are constructed from behaviour through considering notions of low social skills in autism. I focus upon autistic low social skills, rather than social skills more generally, to draw upon rich historical and modern examples. I take it as uncontroversial that people can exhibit a large variety of behaviour in social situations involving two-way interpersonal communication. Additionally, some of that behaviour can fall outside implicitly conceived norms about what is acceptable conduct in social situations (views on what is acceptable conduct can vary between different social groups, cultures and historical eras). All these behaviours are in some sense real and actually occur, just as the ball that Galileo rolled down the inclined plain was real and took a particular path.^2^ In this sense, people exhibit non-constructed behaviour. However, these behaviours are not the same as symptoms because, as described earlier, specific details are missing from the abstract symptom. I highlight this with an historical example of alternative conceptualisations that illustrates that scientists can construct the symptom ‘low social skills in autism’ in different ways.

Understandings of ‘low social skills’ in autism have varied from the 1940s to the current day. During the 1940s to the 1960s, an era when psychoanalysis held significant influence, psychiatrists placed great significance on the subjective emotional life of individuals. They believed that emotional processes could significantly influence which symptoms were exhibited and how they were exhibited (Evans 2017, chapter 5; Hollin 2014, p. 104; Nadesan 2005, chapter 5; Silverman 2012, chapter 3). Many child psychiatrists believed the symptoms of autism, including low social skills, were caused by underlying emotional processes. Interest in cognition as an alternative understanding of the mind started around the 1960s and by the early 1980s focus on cognitive processes had largely replaced interest in emotional processes. Consequently, the low social skills of autism came to be re-conceptualised as resulting from cognitive abnormalities such as theory of mind deficits rather than as a reaction to inner emotional processes (Evans 2017, chapter 5; Eyal et al. 2010, chapter 9; Hollin 2014, p. 5; Verhoeff 2013, p. 451). I will show that varying understandings of low social skills can result in the symptom being constructed in different ways. We can take it as incontestable that cognition and emotions can both potentially influence whether someone struggles in social situations, but depending on the relative importance attached to these factors, the symptom of autistic low social skills can come to be formulated in four alternative ways.

Firstly, autistic low social skills could be considered fundamentally cognitive in nature. They would be considered to be caused by one or more of the main cognitive psychological theories of autism (theory of mind deficits, weak central coherence or executive dysfunctions (see Hill and Frith 2003)). On such an approach it makes sense to abstract away the influence of emotions when formulating the symptom because emotional reactions would be considered incidental. Emotions would be one of many factors which influence how and when symptoms are exhibited. Just as who is being spoken to can be an influence yet is not actually the cause of low social skills, so too emotions would be seen as an influence but not the cause of low social skills. Consequently, they could be abstracted away. For example, on this approach an individual who struggled in a social situation because they were anxious or experiencing emotional turmoil would not then be considered to have the symptom autistic low social skills. Similarly, the way an autistic individual exhibits low social skills might be influenced by emotional factors yet they are only incidental causes and thus not the actual cause of low social skills. This is the general approach adopted by contemporary psychiatry when formulating the symptom of autistic low social skills (see Baron-Cohen 2001; Happe and Frith 2006; Hill 2004). An individual who was believed to exhibit low social skills because they were in distress rather than because they had abnormal cognition would not be considered to actually have autism by most psychiatrists or psychologists (Hollin 2014, p. 13). For example, a leading British researcher on autism studied Romanian orphanage children who had undergone severe sensory and emotional deprivation. Though many met the diagnostic criteria for autism he considered them to have ‘quasi-autism’ (Evans 2013, p. 23; Evans 2017, p. 419; Hobson 2002, p. 203).^3^

Secondly, autistic low social skills might be considered fundamentally emotional in nature. This would work in a similar way to the first example except that rather than emotions being abstracted away, cognitive factors would instead be abstracted away. Low social skills would be considered emotional in nature whereas cognitive factors are incidental influences which can be abstracted away. This approach has some similarities to notions of low social skills employed in relation to autism during the 1930s to 1970s. Due to biological or environmental factors (or both), an individual who inadequately integrated their psychology with their external environment could face additional stresses from the environment or receive inadequate levels of comfort. This led to the individual developing defence mechanisms to protect themselves, and those defence mechanisms caused the symptoms (Bender 1953, p. 667). During this period some child psychiatrists believed autistic children could have both low social skills and low intellect but conceptualised the low social skills as stemming from anxiety whilst low intellect was considered a confounding factor which did not itself cause low social skills (Bender 1959, p. 82–84; Creak 1963, p.88; Eisenburg 1956, p. 21).^4^

Thirdly, both cognition and emotions could be considered central, non-incidental causal factors. Low social skills could be caused by either emotions or cognition or both simultaneously. On such an approach a particular behaviour is an instance of low social skills so long as at least one of these causes is present. Many factors, such as who is being spoken to and for what reason, would be considered incidental but neither emotions nor cognition would be. Some commentators have similarly argued that there are both cognitive and emotional aspects to low social skills in autism and that both, whether occurring together or alone, are manifestation of autistic low social skills (Hobson 2002, p. 134; Hodge 2004, p. 53; Maiese 2013, p. 181).

Fourthly, the symptom low social skills could be split up into two different symptoms. One symptom, cognitive low social skills, would be caused by cognition whilst a different symptom, emotional low social skills, would be caused by emotions. All other factors would be considered incidental and thus abstracted from the generalised symptom.

These alternative ways of formulating low social skills shows that symptoms are constructed by considering two causal factors (I have purposefully only focused on two factors; in reality many other causal factors can influence low social skills and this further emphasises that symptoms are constructed). This had a significant influence over how symptoms are understood. For example, historian of autism Bonnie Evans describes the move from psychoanalytical understanding of autism, which primarily focused upon emotional states, towards cognitive approaches as being a “radical transformation” (Evans 2017, p. 240). Psychiatry had become much more impersonal. Focus shifted away from the subjective feelings and specific interpersonal relationships of particular patients towards supposedly objective cognitive mechanisms. This meant that what counted as low social skills, and how low social skills was investigated, change significantly (this was also influenced by other changes which occurred during this time period, such as the move from case studies based upon clinical reports towards psychological experiments and statistical analysis (Evans 2017, p. 211)). I have shown that choices need be made over which instances of behaviour and which causal factors to abstract away. Low social skills are not ready-made in one of those ways. Rather, symptoms are constructed through a process of abstracting away behaviour and causal factors. In the remainder of this article, I shall firstly consider constraints on how symptoms are formulated and then consider the epistemic consequences of seeing symptoms as constructed.

## Constraints on symptom formulation

I now show how, although there are choices to be made when symptoms are formulated, there are significant constraints on these choices. These are important because construction is often associated with arbitrariness. However, as I show in the subsequent section, these constraints mean that symptoms should not be understood in such epistemologically negative ways. For now, I will outline three different constraints.

Firstly, symptom formulation is constrained by limiting the number of causal factors which are considered non-incidental. It is not practically possible to account for every causal factor so some will be considered as incidental. As previously described, scientists typically aim to isolate and control causal factors because only some are considered relevant to the phenomena under study. Scientists will aim to prevent some causal factors occurring whilst trying to make other causal factors occur. Some philosophers believe that which factors should be considered relevant is dependent upon theory (Massimi 2007, p. 249; Woodward 2010, p. 797; Teller 2010, p. 824; Psillos 2004, p. 397) or at least reliant upon non-empirical considerations (Bogen 2010, p. 779; Glymour 2000, p. 32; Woodward 2010, p. 797). The melting point of lead is often considered to be posited on theoretical grounds because 235° is the figure molecular theory predicts once incidental factors are abstracted away. This involves theoretical assumptions, such as the nature of chemical bonding. Similarly, to demarcate a behaviour as being caused by social understanding rather than emotional or intellectual understanding involves many theoretical assumptions about the functioning of the mind, about human communication and about how humans relate to their environment. In relation to autism, low social skills is currently considered to be caused by cognitive differences like theory of mind deficits. Consequently, it seems uncontroversial that an experiment measuring autistic low social skills should exclude an individual who was drunk. Alcohol intoxication may result in behaviour which can resemble instances of low social skills but that drunken behaviour does not constitute low social skills because the behaviour was caused by non-relevant causes. Similarly, an individual who had a history of emotional neglect may also exhibit behaviour which resembles low social skills but that behaviour would be considered quite different to autistic low social skills because it was caused by childhood experiences rather than a lack of theory of mind (see Evans 2013, p. 23; Evans 2017, p. 419 for analysis).^5^

Secondly, symptom formulation is also constrained by the need for generality. Phenomena are formulated at a greater level of generality than are data. They are not tied to specific instances of data, rather, they cover multiple instances of data so are formulated in an abstract and generalised manner (Bogen & Woodward 1988, p. 324). Formulating a phenomena at too specific a level of detail means that there is often less tractability (Batterman 2002, p. 22), reduces generality (Rohwer and Rice 2013, p. 336) and applicability to future situations (Myrvold and Harper 2002, p. 137). Symptoms are also things which can occur in multiple contexts and are not tied to one particular context. Low social skills, for example, can occur in multiple contexts (home, school, work), be exhibited towards multiple classes of people (lifelong friends, complete strangers, colleagues), and for multiple reasons (casual conversation, requesting information, arguing points). It is implausible that a different notion of low social skills could be formulated for each of these (and many other factors). Having many context-dependent symptoms would create immense practical problems, such as massively increasing the number of symptoms mental health professionals would need be trained about and consider when assessing patients. Additionally, whilst it is possible to control for many factors during experiments there will be some factors that it is near impossible to control for experimentally such as many aspects of an individual’s unique personality and past history. Consequently, it will be impossible to fully investigate a symptom independent of many factors which can influence how instances of behaviour can manifest.

Thirdly, phenomena need be constrained by accuracy. The phenomena need be representative of the data. Phenomena can track the data to a varying degree of accuracy. How much accuracy is considered acceptable depends upon the goals of the scientists (Batterman 2002, p. 37; Teller 2010, p. 890; Woodward 2011, p. 174). Whilst phenomena will not accurately portray all, or even any, of the data it need be constrained by the data. It need not be fully accurate for two reasons. Firstly, it can be legitimate to disregard instances of data which fall sufficiently outside expected results (Woodward 2000, p. 177). Additionally, the data which is considered relevant will then be heavily idealised when the phenomena is formulated. For example, variance in data points will be smoothed out when a graph curve is charted when a data model is produced. Despite this, phenomena still needs to be constrained by the data. It would be illegitimate for scientists to simply pick and choose which data to ignore. Rather, there would need to be reason to believe the apparatus was not functioning correctly, or that the environmental variables are too dissimilar to other relevant experiments, or that the results fall outside a range predicted by a well established theory. Similarly, scientists cannot idealise the data in any manner that they choose. A scientist cannot simply posit a curve on a graph in any place they feel like doing so. Rather, they are constrained by various sophisticated mathematical, statistical and modelling techniques (Woodward 2000, p. 145). None of these determine which phenomena scientists formulate but they do constrain which phenomena scientists can legitimately formulate.

The three factors I have outlined show that there are significant constraints on symptom construction. This means that symptom formulation is not a purely arbitrary process. These constraints do not determine which symptoms are formulated because choices need be made when these constraints are applied. Nothing in the world determines which causes to abstract away or how theories should be formulated, rather, these depend upon the weighing of theoretical virtues (Psillos 1999, p. 171; Solomon, 2001 pp. 19–20; Day and Kincaird 1994, p. 275). Similarly, nothing in the world determines what degree of accuracy and what degree of generality is required for a symptom to be appropriately constructed, rather, choices need be made. Although choices need be made when formulating symptoms the choices are constrained choices and thus not purely arbitrary ones.

## The epistemic status of symptoms

Many critics who are sceptical about psychiatric diagnoses do not extend their scepticism to psychiatric symptoms. However, given my demarcation between behaviour and symptoms, I will now show that criticisms against psychiatric diagnoses which stem from concerns about construction are also applicable to symptoms. It is worth noting that I am not myself endorsing these criticisms of psychiatric diagnoses. Rather, I suggest that those who do endorse these criticisms in relation to psychiatric diagnoses may have to endorse them in relation to symptoms also.

Firstly, psychiatric diagnoses typically cover heterogeneous groupings of symptoms (Kozak and Cuthbert 2016, p. 287; Sanislow et al. 2010, p. 632). The symptoms exhibited by two people with the same diagnosis can differ significantly. This is taken to show that diagnoses tell us little about actual people (Smith and Combs 2010, p. 210). However parallel worries can be raised about symptoms. Symptoms typically cover heterogeneous groupings of behaviour. Two instances of behaviour may be instances of the same symptom yet differ from one another quite significantly. For example, an individual could be considered to exhibit low social skills by not taking part in conversations, by abnormal body language, by speaking over people, by monologuing, by being rude, by not respecting typical flow of conversation, by being confrontational, etc. Additionally, consider how auditory hallucinations can take the form of people who the individual knows, people who they do not know, robotic voices, alien beings, etc.

Secondly, in many cases numerous, often contestable, judgements have to be made when diagnostic criteria are constructed. For two different reasons there is usually ambiguity and a significant level of choice over how to formulate diagnoses. The first reason is the question of how to conceptualise a psychiatric diagnosis. This often involves emphasising some aspects of the psychiatric diagnosis, seeing them as more central than others. I previously gave the example of how historically the psychiatric diagnosis of autism has been conceived in different ways depending upon whether emotional or cognitive aspects were emphasised.^6^ Similar concerns are true of symptoms. There are also choices in how they can be formulated. Symptoms can be formulated to cover a wider range of behaviours or a narrower range of behaviours (Fellowes 2017, 285; Ochs et al. 2004, p. 155; Woodward 2008, p. 173) and this will influence which symptom a behaviour is assigned to. For example, imagine an autistic person spends a lot of time computer programming in their spare time. This could be understood as an instance of the symptom of obsessive, narrowly focused interests. Alternatively, it could be understood as routine like repetitive behaviour. It also might be understood as an attempt to keep their environment relatively static and thus avoid unexpected changes. It could even be construed as a normal interest which is not a symptom. Which symptom (if any) the behaviour is assigned to depends upon the way in which each symptom is formulated. Additionally, psychiatrists might decide they could better accommodate the behaviour of autistic people by replacing those three symptoms with a much more general symptom, or they could split each symptom into multiple, more specific symptoms. The second reason why there is ambiguity and choice is that psychiatric diagnoses cover correlations of symptoms. That is, clusters of symptoms which are likely to occur together in people with the diagnosis. However, symptoms typically only cluster together very weakly. Symptoms do not cluster in a manner whereby there are clear gaps between symptom clusters. There is significant overlap between clusters. Typically many symptoms do not obviously fall into one cluster rather than another (Kendell and Jablensky 2003, p. 6; Zachar 2014, p. 113). This manner of formulating diagnoses does not, independent of further choices, determine which psychiatric diagnoses are formulated (Kendler et al. 2011, p. 1149; Kincaid 2014, p. 151; Jablensky 2008, p. 90). The computer programming example shows that behaviours do not fall into neatly demarcated clusters. Rather, clusters of behaviour can overlap. Some behaviours will cluster together as more stereotypical instances of obsessive, narrowly focused interests (such as intense knowledge of an obscure topic and ability to monologue in conversations) and other behaviours will cluster together as more stereotypical instances of routine like repetitive behaviour (such as repetitive physical movements and keeping to schedules) but the computer programming example shows a behaviour which falls between these clusters. This creates uncertainty over which symptom the behaviour should be assigned to.

Thirdly, psychiatric diagnoses appear to typically cover causally heterogeneous individuals. The causes present in one individual with a diagnoses can vary significantly from those present in another individual with the same diagnosis (Cuthbert and Insel 2013, p. 3; Kozak and Cuthbert 2016, p. 287; Sanislow et al. 2010, p. 632). This means that psychiatric diagnoses typically only correlate with causes in a very weak manner and that psychiatrists cannot (or so far have failed to) demarcate psychiatric diagnoses based upon causes. The same is true of many symptoms. The causal influence on a particular instance of behaviour can be quite different to the causal influences on a different instance yet both instances of behaviour can constitute instances of the same symptom. Recent evidence suggest that there is no direct relationship between causes and symptoms. The same symptom can arise from multiple causes whilst the same cause can result in multiple symptoms (Cloninger 2014, p. 205; Kendler 2005, p. 1247). For example, Murphy outlines how instances of delusion appear to have very diverse causal underpinnings (2014, p. 120). This shows that multiple causes can produce the same symptom. Hoffman and Zachar outlines how the amygdala is causally connected to phobias, anxiety, disgust and decision making (2017, p. 70). This shows how the same cause can produce multiple symptoms. Relatedly, there are concerns that attempts to causally demarcate psychiatric diagnoses will result in focusing on only some causes at the expense of other causes. For example, demarcating a diagnosis on biological factors typically means idealising away the influence of psychological factors. Additionally, there might be choices over which causes to focus upon (Kendler et al. 2011, p. 1149; Philips 2015, p. 170; Poland 2014, p. 35). Similarly, constructing symptoms from behaviour requires idealising away many causal factors. Only some will be considered relevant to the symptom and this will involve a level of choice. As I outlined earlier, autistic low social skills is typically understood to stem from cognitive abnormalities but social and emotional factors can also play a major causal role in when and how autistic people understand social information. It would be difficult to causally demarcate autistic low social skills upon all relevant causal factors.

Fourthly, psychiatric diagnoses are typically considered to be disorders. The concept of disorder is a heavily debated area by philosophers of medicine. There are multiple models of disorder and there is little consensus upon which is correct (for example, see Cooper 2007; Wakefield 1992). This can lead to disagreement over whether a particular diagnosis is a legitimate disorder (for example, Potter (2013) in relation to oppositional defiance disorder, see Wakefield (2012) in relation to depression). Also, there can be borderline cases where it is unclear if someone with only mild symptoms is actually disordered (see Horwitz in relation to depression (2014) and Timini et al. (2011) in relation to autism). These problems are also applicable to symptoms. It can be unclear whether any particular symptom should be considered pathological or when a particular behaviour should be considered an instance of a pathological symptom. For example, autistic people often exhibit repetitive, stereotyped behaviour known as stimming. These are typically considered to be pathological symptoms but at least in some instances, they cause no harm to the individual or anyone else. They may actually be a helpful means of coping with anxiety (Doan and Fenton 2013, p. 61). Similarly, there is debate over whether all, or only some, instances of hallucinations should be considered to be pathological. Some people do not seem impaired by hallucinations and it is unclear where the cut off point between pathological and non-pathological hallucinations should be placed (Bentall 2014, p. 40). There is also debate over whether some types of hallucinatory content, such as religious content, should be considered pathological or non-pathological (Rashed 2010, p. 189).

## Reappraising diagnoses

I have shown that some critics who see most or all psychiatric diagnoses as problematic constructs have similar concerns over few or no symptoms. However, I have so far made the following claims: (1) symptoms are constructed, (2) there are non-arbitrary constraints on symptom formulation, (3) arguments which critics use against psychiatric diagnoses are also applicable to symptoms, including against symptoms which should be considered non-arbitrary. If all these three points are accepted then I suggest that there are three possible options available to the critics of psychiatric diagnoses.

Firstly, since both symptoms and psychiatric diagnoses are constructed, and since construction is considered a reason for concern over diagnoses, critics of diagnoses might extend their concern to symptoms. They could claim that currently employed symptoms are constructions and in need of replacement with superior, non-constructed symptoms. Alternatively, they may believe that currently employed symptoms should not simply be replaced with better formulated symptoms, rather, they should be replaced with an alternative way of understanding people which does not involve symptoms. On this reading, the four concerns that are held over psychiatric diagnoses described in the previous section, which I argued were also applicable to symptoms, would be considered fatal to both psychiatric diagnoses and symptoms. This would be true regardless of any non-arbitrary constraints on symptom formulation.

Secondly, a critic of psychiatric diagnoses might remain relatively unconcerned about symptoms even though symptoms are constructed. This would mean the critic would have reason to not automatically link construction with epistemic concerns. Consequently, that something is constructed is not then reason to have epistemic doubts over it. This then undermines a major motive for epistemic doubts over diagnoses and consequently could undermine calls to replace diagnoses. Critics of diagnoses who considered symptoms to be scientifically legitimate would have reason to no longer reject psychiatric diagnoses. On this reading, the four concerns which critics have of psychiatric diagnoses would no longer be considered epistemologically problematic, either for psychiatric diagnoses or for symptoms.

Thirdly, a critic could attempt to supply an argument which showed that symptoms are constructed in an unproblematic way whereas diagnoses are constructed in a problematic way. Therefore, there would be reason to reject diagnoses without rejecting symptoms. The viability of this approach will depend upon further argumentation from these critics. Some psychiatric diagnoses may be constructed in a problematic manner and some may be constructed in an unproblematic manner. A critic who accepted that construction is not automatically reason to reject psychiatric diagnoses would need give specific reasons to reject particular psychiatric diagnoses. On this reading, those four concerns over psychiatric diagnoses which critics have would still be considered problematic for psychiatric diagnoses but not for symptoms. One possible approach would be to argue that the three constraints on symptom formulation leave symptoms non-arbitrary but that there are no similar constraints on psychiatric diagnoses.

The primary aim of this paper is to argue that diagnoses and symptoms are both constructed. Those who hold that diagnoses are constructed, should accept that symptoms are constructed too. This paper poses a challenge to those critics of psychiatry who think that psychiatric diagnoses are constructed, and so problematic, but who take symptoms for granted. I have shown that such a stance is untenable. I have sketched three options for critics of psychiatric diagnosis. To end, I will briefly set out some of my reasons for preferring the second option – i.e. accept that both diagnoses and symptoms can be constructed and also legitimate—by drawing upon Psillos’ realist notion and Massimi’s neo-Kantian notion. Psillos bases realism on whether a theory contributes to the best explanatory framework for understanding the world (2011, p. 15). Massimi describes how entities are “functionally relevant clusters of properties” (2014, p. 428) which should be projectable (inductive) and should be embedded within a wider network of claims (2011, p. 110; 2014, p. 428). Psychiatric diagnoses can potentially play these roles.

A psychiatric diagnosis is an abstract entity which groups together people who exhibit at least a certain number of symptoms of a diagnostic criteria. Rather than simply considering a particular individual with particular characteristics, the abstract psychiatric diagnoses abstracts away many significant aspects of particular individuals but then draws commonalities between many particular individuals. This means that psychiatric diagnoses can enhance our understanding of people *at a particular level of abstraction*.

This helps understanding the world in two ways. Firstly, it assists inductive claims. Knowing that an individual accurately fits the diagnostic criteria of autism allows many probabilistic inductive claims of how particular individuals act in particular situations. Whilst most psychiatric diagnoses cover individuals with heterogeneous symptoms this still allows a significant level of non-trivial inductions. Despite the level of heterogeneity of associated symptoms, knowing someone is autistic, schizophrenic or fails to meet the diagnostic criteria of any psychiatric diagnoses provides significant information about possible behaviour. This provides inductive knowledge at a level of abstraction which is not present when considering particular individuals.

Symptoms can also assist inductive claims. Knowing someone has the symptom low social skills entails probabilistic inductive claims about possible manifestations of behaviour in a variety of settings. This is true even though a symptom can cover a wide range of behaviour.

Secondly, psychiatric diagnoses can be embedded within a wider framework of knowledge. They can be embedded within a network of probabilistic causal factors and within theoretical accounts of how the mind works and how humans relate to one another. This systematically creates links between different parts of the world which can also be used for explanatory claims at a particular level of abstraction. Rather than considering how a particular individual relates to these factors, a psychiatric diagnosis shows how a generalised, abstract class relates to these factors. This is helpful because causes (be them genetic, neurological, psychological or environmental) in psychiatry are almost always linked to symptoms and diagnoses in a probabilistic manner. Causation often requires combinations of many different factors working together, and changes in the combinations of factors sometimes will result in a different outcome (or no outcome) and sometimes will not (Cuthbert and Insel 2009, p. 989; Haslam 2002, p. 209; Lilienfeld 2014, p. 136). Also, causes sometimes take the form of a developmental pathway where a series of processes will unfold which can depend upon the specific process present in the earlier chain of events and which can vary from case to case (Casey et al. 2014, p. 351). That differing combinations of causal factors have different probabilities of outcomes is not highlighted by just considering one specific individual with one specific set of causes. In contrast, the way in which probabilities combine together based upon the presence of various causal factors is highlighted by considering multiple individuals, with various combinations of causes, to constitute instances of an abstract psychiatric diagnoses (see Schaffner’s diagram on the probabilities of developing depression for an example (2008, p. 54)).

Symptoms can also be embedded within a wider framework. They are embedded into psychiatric diagnoses and can also be embedded within a network of probabilistic causal factors. Additionally, symptoms can be used to embed behaviour. Rather than simply considering each instance of behaviour as being unique, particular instances of behaviour can be embedded within symptoms. A particular behaviour is not simply a unique instance of social interaction, rather, it can fall into the general symptom low social skills. This means unique instances of behaviour can be embedded within abstract symptoms which can then be embedded within psychiatric diagnoses and probabilistic causal factors.^7^

This shows that psychiatric diagnoses are worth employing because they can provide useful information at a certain level of abstraction. However, this is not to claim that other ways of abstraction are unimportant. There are three types of alternative ways of abstraction. Firstly, someone can be considered an instance of a psychiatric diagnosis *and* be considered a unique individual. The psychiatric diagnosis relates the individual to a general class whereas it will also be useful to focus on the individual to describe many aspects not described by their psychiatric diagnosis. Secondly, I have focused upon categorical, polythetic psychiatric diagnoses. There are also dimensional and psychodynamic diagnoses and these would provide information at other levels of abstraction. It will often be useful to employ multiple types of psychiatric diagnoses simultaneously to provide information in various abstract ways. Thirdly, alternative categorical polythetic psychiatric diagnoses which involve different abstract groupings might be superior to currently employed ones. This is certainly a possibility but they would still be constructed and cover heterogeneous symptoms and causes. The possibility of superior categorical, polythetic psychiatric diagnoses does not mean current ones should be dismissed.

## Conclusion

In this paper I have developed a new conceptual understanding of symptoms. I have drawn upon the distinction between data and phenomena to demarcate between behaviour and symptoms. Behaviour is what people exhibit. Symptoms are how psychiatrists model that behaviour. This means that symptoms are constructed rather than being pre-built parts of the world. I have outlined three different non-arbitary constraints on constructing symptoms, namely causal relevancy, frequency and generality.

Critics of psychiatric diagnoses are typically not concerned about psychiatric symptoms. Much of the concern over psychiatric diagnoses stem from them being constructed. However, I have shown that symptoms are also constructed. Additionally, I have shown that common criticisms of psychiatric diagnoses are also applicable to symptoms. To finish I sketched my preferred response to the dilemma I have posed; I suggest that both symptoms and psychiatric diagnoses can be of scientific and epistemological value despite being constructed.

## Footnotes

1 I will use the word symptom in a manner which glosses over a terminological distinction which is sometimes employed in medicine. Sometimes, a distinction is made between signs, which are observable by an external agent, and symptoms, which are unobservable feelings of the illness. However, philosophers of psychiatry rarely make a distinction between signs and symptoms. I also think there is little philosophical significance to be placed on the distinction because, even though one is observable, both are theory-laden. Also, the terminology of signs and symptoms does not match how people typically talk of symptoms. For example, low social skills is typically described as a symptom of autism even though it is strictly speaking composed of both signs and symptoms. Consequently, I will lump signs and symptoms together into a general notion of symptoms.

2 It might be more accurate to say that the ball that Galileo rolled down the inclined plain was real and took a particular path when measured by creatures with our sensory capacities. So too the behaviour is actually real when measured by creatures with our sensory capacities and our (potentially culturally specific) social norms.

3 As they aged many would lose the symptoms of autism though by adulthood a significantly larger portion still met the diagnostic criteria compared to typical prevalence rates.

4 Low intellect is not the same as cognition but it is sufficiently similar to highlight my point. Low intellect could influence how a child expressed low social skills and so was considered a confounding factor when studying low social skills. It was not, however, considered to be the cause of low social skills with autistic children. Also, note that children in these decades who exhibited a clinical picture associated with modern notions of autism were often diagnosed under different names such as childhood schizophrenia.

5 However, some critics would argue that demarcation is made on questionable theoretical grounds and that autistic low social skills could arise purely from childhood experiences (Hobson 2002, p.203).

6 I thank an anonymous reviewer for pushing me to clarify this paragraph. In doing so they provided helpful additional examples of different ways to conceptualise psychiatric diagnoses, such as how gambling disorder could be considered an impulsion, an obsession or an addiction. They also mentioned that researchers emphasise the importance of different symptoms when conceptualising long term, low grade depression depending upon whether or not they think it is a personality disorder.

7 It could be responded that I have not met Psillos’ criteria of an *explanatory* framework. However, probabilistic causation is still explanatory. It explains how a large number of factors can combine together to produce various effects in particular individuals. Additionally, a psychiatric diagnosis can explain symptoms much like how Psillos describes how a more abstract model, such as a Linear Harmonic Oscillator, can explain a less abstract model, such as a specific spring. Psillos argues the relationship can be explanatory despite not being causal (2010, p. 957). In this manner an abstract diagnosis can explain the symptoms of a particular individual despite not causing them.
